# Review of "Keys to the Nematode Parasites of Vertebrates. Archival Volume" by Roy C. Anderson, Alain G. Chabaud and Sheila Willmott (eds.)

**DOI:** 10.1186/1756-3305-2-42

**Published:** 2009-09-17

**Authors:** František Moravec

**Affiliations:** 1 Institute of Parasitology, Biology Centre of the Academy of Sciences of the Czech Republic, Branišovská 31, 370 05 České Budějovice, Czech Republic

## Abstract

Book review of "Keys to the Nematode Parasites of Vertebrates. Archival Volume" by Roy C. Anderson, Alain G. Chabaud and Sheila Willmott (eds.)

## Review

The identification of parasitic nematodes, members of an extremely large animal phylum, has never been easy and, in fact, only three monographs with keys to all these parasites were available in the second half of the last century. A four-volume "Key to Parasitic Nematodes" was published in Russian by Skryabin et al. (1949-1954), being accompanied by a 29-volume series "Essentials of Nematology" of then Soviet authors (1949-1977), and a two-volume "Systema Helminthum III. The Nematodes of Vertebrates" by Yamaguti (1961). However, for many years, mainly "CIH Keys to the Nematode Parasites of Vertebrates", published by Anderson, Chabaud and Willmott in ten parts between 1974 and 1983, have served as an absolutely essential working tool for the generic identification of nematodes parasitic in vertebrates. The same taxonomic arrangement was later followed in the comprehensive book by Roy C. Anderson, now in its second edition (Nematode Parasites of Vertebrates: Their Development and Transmission. CAB International 2000), representing a unique, invaluable overview of nematode life cycles. Due to popular demand and to coincide the publication of the supplement volume, a re-publication of the refreshed Keys with reordered superfamilies in a single volume, is now available.

The classification system and all texts and illustrations (line drawings) of this volume are exactly the same as in the first edition of the Keys, including the designation of Nematoda as a class, only the superfamilies are now arranged in a systematic order. It is explained in the Foreword to this volume by O. Bain, I. Beveridge, M.-C. Durette-Desset and A. Chabaud that recent molecular studies have not changed greatly the groupings among the parasitic taxa of nematodes (now considered a phylum), although there are some small discrepancies, so that it is not necessary to abandon the earlier classification used in the Keys, which was based on morphological and biological features.

The text of the volume is divided into 15 chapters, which were written by well-known international experts, nine leading scientists from Canada (Roy C. Anderson), France (Odile Bain, Alain G. Chabaud, Marie-Claude Durette-Desset, Annie J. Petter and Jean-Claude Quentin), Germany (Gerhard Hartwich), UK (Sheila Willmott) and USA (J. Ralph Lichtensfeld). After the first chapter providing a very useful glossary and keys to subclasses, orders and superfamilies (17 pages), the following 13 chapters (431 pages) deal with individual orders Enoplida, Rhabditida, Strongylida, Oxyurida, Ascaridida and Spirurida and form the core of the book, covering 770 nematode genera belonging to 89 families and 27 superfamilies. Each of these chapters includes authors' short taxonomic remarks to the higher taxa in the respective superfamilies, followed by the dichotomous keys to families, subfamilies, genera and subgenera (including synonyms), and references to the most relevant literature. The index (pp. 449-463) includes page numbers to all taxa mentioned in the book, irrespective of synonymization, which enables much better orientation in the text; unfortunately, this was absent in the first edition of the Keys. Generally, the quality of line drawings illustrating the main taxonomic features is good, except for a few in the second chapter (Figs. Nine.31, Nine.34, Nine.35, Nine.38, Nine.41-Nine.43), where the originals were probably too reduced. In contrast to the "Keys to the Cestode Parasites of Vertebrates" by Khalil et al. (1994) and the three-volume "Keys to the Trematoda" by Gibson et al. (2002), Jones et al. (2005) and Bray et al. (2008), recently published by CABI (the latter in a coedition with The Natural History Museum, London), this volume does not contain diagnoses of the genera and higher taxa, as in the first edition of the Keys.

It is clear that due to continuing parasitological studies, often carried out on materials originating from previously not or little explored geographical regions or hosts, many new forms of parasitic nematodes are gradually discovered and described. Consequently, every comprehensive monograph treating the taxonomy of these parasites as a whole becomes quickly out of date. It is the reason why a new, supplementary volume of the Keys, written by Lynda M. Gibbons, will appear soon in CABI, covering some 300 additional nematode genera described since the first edition. Because the classification systems of some nematode groups (e.g., Dracunculoidea or Trichinelloidea) as given in the archival volume of the Keys have been later changed, both archival and supplementary volumes should be used together.

The present re-issue of the Keys, 35 years since their first publication, can be highly appreciated, because, as stated in the Foreword to this volume, there is a persistent demand and need for means to identify parasitic nematodes as well as a resurgent interest in the origins and evolution of parasitism within the phylum. It may also help to link recent molecular insights with those provided to date in numerous morphological studies, and to establish links between studies on animal-parasitic and free-living nematodes. Undoubtedly, this excellent monograph represents one of the basic works on parasitic nematodes of vertebrates, which may also become a basis for subsequent revisions of taxonomy as well as for studies on the biology, ecology, zoogeography, etc. of these parasites. This will certainly be appreciated as an important source of information and an indispensable tool for the determination of nematodes not only by parasitologists, but also interested physicians, veterinarians, zoologists, wildlife and fisheries biologists, university students and others. The authors, editors and the publishers are to be congratulated on this publication.

## Competing interests

The author declares that they have no competing interests.

